# Effect of traditional yoga, mindfulness–based cognitive therapy, and cognitive behavioral therapy, on health related quality of life: a randomized controlled trial on patients on sick leave because of burnout

**DOI:** 10.1186/s12906-018-2141-9

**Published:** 2018-03-06

**Authors:** Astrid Grensman, Bikash Dev Acharya, Per Wändell, Gunnar H. Nilsson, Torkel Falkenberg, Örjan Sundin, Sigbritt Werner

**Affiliations:** 1 Department of Neurobiology, Care Sciences and Society, Division of Family Medicine and Primary Care, Karolinska Institutet, Alfred Nobels allé 23, 141 83 Stockholm, Sweden; 2 Department of Neurobiology Care Sciences and Society, Division of Nursing, Research Group Integrative Care, Karolinska Institutet, Stockholm, Sweden; 3 Centre for Social Sustainability, Karolinska Institutet, Stockholm, Sweden; 4 Faculty of Human Sciences, Department of Social Sciences, Mid Sweden University, Östersund, Sweden; 5 Department of Medicine, Karolinska Institutet, Stockholm, Sweden

**Keywords:** Stress-related disorder, Burnout, Exhaustion syndrome, Work-related stress, Randomized controlled trial, Traditional yoga, Mindfulness-based cognitive therapy, Cognitive behavioral therapy, Mind-body therapies, Integrative medicine

## Abstract

**Background:**

To explore if health related quality of life(HRQoL) increased after traditional yoga(TY), mindfulness based cognitive therapy(MBCT), or cognitive behavioral therapy(CBT), in patients on sick leave because of burnout.

**Methods:**

Randomized controlled trial, blinded, in ninety-four primary health care patients, block randomized to TY, MBCT or CBT (active control) between September 2007 and November 2009. Patients were living in the Stockholm metropolitan area, Sweden, were aged 18–65 years and were on 50%–100% sick leave. A group treatment for 20 weeks, three hours per week, with homework four hours per week. HRQoL was measured by the SWED-QUAL questionnaire, comprising 67 items grouped into 13 subscales, each with a separate index, and scores from 0 (worse) to 100 (best). SWED-QUAL covers aspects of physical and emotional well-being, cognitive function, sleep, general health and social and sexual functioning. Statistics: Wilcoxon’s rank sum and Wilcoxon’s sign rank tests, Bonett-Price for medians and confidence intervals, and Cohen’s D.

**Results:**

Twenty-six patients in the TY (21 women), and 27 patients in both the MBCT (24 women) and in the CBT (25 women), were analyzed. Ten subscales in TY and seven subscales in MBCT and CBT showed improvements, *p* < 0.05, in several of the main domains affected in burnout, e.g. emotional well-being, physical well-being, cognitive function and sleep. The median improvement ranged from 0 to 27 points in TY, from 4 to 25 points in CBT and from 0 to 25 points in MBCT. The effect size was mainly medium or large. Comparison of treatments showed no statistical differences, but better effect (small) of both TY and MBCT compared to CBT. When comparing the effect of TY and MBCT, both showed a better effect (small) in two subscales each.

**Conclusions:**

A 20 week group treatment with TY, CBT or MBCT had equal effects on HRQoL, and particularly on main domains affected in burnout. This indicates that TY, MBCT and CBT can be used as both treatment and prevention, to improve HRQoL in patients on sick leave because of burnout, reducing the risk of future morbidity.

**Trial registration:**

July 22, 2012, retrospectively registered. ClinicalTrails.gov NCT01168661. Funding: Stockholm County Council, grant 2003–5.

## Background

Burnout and other mental health problems related to stress have increased in prevalence in recent decades and currently constitute a substantial problem worldwide with escalating costs [1, 2]. The cause of burnout is thought to be related to work and, to some extent, the individual’s personal situation [3, 4]. It is characterized by emotional and physical exhaustion [3–5]. No common definition of burnout exists, but the Maslach definition and the Maslach Burnout Inventory are often used in research [3, 6]. Emotional and physical exhaustion are the core symptoms of Exhaustion Syndrome, which has been used in Sweden since 2005 to diagnose burnout in a medical context, with the diagnostic code F43.8A in the ICD-10 [7]. The diagnostic criteria are listed in Table 1. The majority of patients diagnosed with burnout are women aged 18–49 years, although the number of men affected is increasing [8, 9]. Though mainly work related, patients are diagnosed and rehabilitated not only in occupational medicine, but also in primary care [10] and other health care settings, such as psychiatry. Depression and anxiety may follow or coexist [9, 11, 12]. Long-term sick leave is common, especially if the condition has lasted for a long time and severe symptoms are present [9]. A long-standing sensitivity to stress often occurs in burnout patients and may postpone full recovery [13]. Relapse is common and there is a risk for cronocity [14, 15]. Considered together, the morbidity and costs are enormous at all levels of society—on the personal level, in the health care system and at companies [2, 16]. At companies the costs may arise from higher levels of absenteeism and sick leave, employee turnover, presenteeism with deterioration of productivity and an increased risk of mistakes and accidents [2, 16].

**Table 1:** Diagnostic criteria for Exhaustion syndrome, ICD-10 code F43.8A

A	Physical and mental symptoms of exhaustion with minimum 2 weeks duration. The symptoms have developed in response to one or more identifiable stressors which have been present for at least 6 months	
B	Markedly reduced mental energy which is manifested by reduced initiative, lack of endurance, or increase of time needed for recovery after mental efforts	
C	At least four of the following symptoms have been present most of the day, nearly every day, during the same 2 week period:	
	1	Persistent complaints of impaired memory
	2	Markedly reduced capacity to tolerate demands or to work under time pressure
	3	Emotional instability or irritability
	4	Insomnia or hypersomnia
	5	Persistent complaints of physical weakness or fatigue
	6	Physical symptoms such as muscular pain, chest pain, palpitations, gastrointestinal problems, vertigo or increased sensitivity to sounds
D	The symptoms cause clinically significant distress or impairment in social, occupational or other important areas of functioning	
E	The symptoms are not due to the direct physiological effects of a substance (e.g. abuse of a drug, a medication) or a general medical condition (e.g. hypothyroidism, diabetes, infectious disease)	
F	The stress-related disorder does not meet the criteria for major depressive disorder, dysthymic disorder or generalized anxiety disorder	

Translation from Glise et al., [9]

Persons with burnout have a lowered quality of life, especially in the emotional sphere [17]. In severe cases such as in our study group, the decrease is global, with low scores also in subscales reflecting sexual function and social interaction [18] and with equal or lower scores than those in chronic somatic diseases and severe psychiatric disorders surveyed in previous research [19–21]. Health related quality of life (HRQoL) has become an increasingly popular measurement in research as it provides us with the patient’s perspective of his or her HRQoL and aids in designing better treatment and prevention [22]. HRQoL also predicts future health and mortality [23], and patients with burnout have been shown to have an increased risk for somatic and psychiatric disease as well as for long-term sick leave [24, 25].

### Rehabilitation for burnout

The rehabilitation recommended is often multimodal with psychological support, usually cognitive behavioral therapy (CBT) in a group or individually as the main component [11, 26], although so far CBT alone has not been shown to have an effect on the length of sick leave, and evidence-based treatment for burnout is not established [27]. Today the length of the treatment in research is usually 8–16 weeks, and the amount of sick leave allowed before inclusion usually does not exceed six months [28]. We do not know what the optimal length of treatment will be for this group of patients, and relapse is a common problem. Other rehabilitation components include support to return to work [14], physical activity and stress management, provided not only in occupational medicine but also in primary care [10]. Few randomized controlled studies have been performed on patients on sick leave with burnout. To find more effective prevention and rehabilitation/treatment, and to decrease the incidence of relapse, a deeper understanding of efficacious treatment components is needed that includes the patient perspective. Methods that can be used in different health care settings are urgently needed. Early intervention and prevention are fairly easily administered, can be less complicated and reduce costs [15]. Both yoga and MBCT are two different methods that have emerged recently in medicine and many patients and health care personnel are interested in these methods and what they can contribute with. Especially when evidence based methods are lacking. CBT is a well documented method which is in this study served as control group. Therapy in group is an advantage as it may lower the costs for therapy, and as there is generally a shortage of therapists. Furthermore, Yoga may be practiced at home with weekly feedback delivered by a yoga instructor under supervision. Also, more optional therapies will increase the availability of therapy and the possibility to find a suitable and individualized therapy.

### Yoga in health care

In recent decades yoga, both traditional and the numerous variations, has become increasingly popular in the West as a means of increasing and maintaining physical, mental and emotional well-being [29]. When reporting on yoga as treatment in RCTs the most common is not to report any specific style. When yoga style is reported, the most commonly used yoga styles in research are Hatha yoga (HY), Iyengar yoga (IY) and Pranayama (breathing exercises) [30]. Yoga has recently been introduced in health care, with programs for stress reduction using for example mindfulness based stress reduction which comprises asanas (physical postures) [31], and yoga has been suggested for rehabilitation after acute myocardial infarction [32]. Surdarshan Kriya Yoga (SKY), has been shown to be effective as a treatment for moderate stress-related disorders*,* and for depression [33]. Asanas (physical postures), breathing exercises and meditation have all shown an effect in different patient groups, as stand alone or more often in a combination. In a meta-analysis of yoga as treatment for major depression SKY and HY for example, were effective [34]. In another meta-analysis HRQoL and mental health in women diagnosed with breast cancer, showed improvement after HY and IY among others [35]. Also, IY has shown an effect in depression [36], to some extent shown an effect in anxiety [37, 38], and as stress-management in healthy [39]. A recent systematic review showed that most yoga styles have the same proportion of positive conclusions, indicating that different yoga styles might have similar effects [40]. The majority of studies in that review had a combination of exercises, such as asanas, breathing exercises and meditation [40]. Traditional Yoga (TY), usually with a combination of these three types of exercises, has an effect both on a physical and an emotional level [41], and could be an interesting treatment alternative in this group of severely sick patients with a global decrease in HRQoL and with diverse symptoms. Few studies have compared HRQoL before and after treatment with TY in severely sick patients with burnout.

### Mindfulness–based cognitive therapy

Mindfulness–based cognitive therapy (MBCT) was developed from translational research to cope with depressive relapse/recurrence [42]. MBCTs theoretical premise is that depressive relapse is associated with the reinstatement of negative modes of thinking and feeling that contribute to recurrence and relapse. MBCT targets cognitive reactivation [42], and the mindfulness component helps the patient to accept aversive thoughts and emotions and adapt an intentionally open, receptive, and flexible disposition with respect to moment-to-moment experience. The effect of MBCT in treating burnout is yet to be established, and, to the best of our knowledge, there is no study addressing this issue.

### Aims

The objectives were to assess the effects of long (20 weeks) treatment with TY, MBCT and CBT (active control) on HRQoL in patients on sick leave because of burnout. The specific aims were: 1) to evaluate the group treatment effect by comparing the scores before and after treatment within each group; and 2) to compare the treatment effects in TY and in MBCT with those of CBT. Finally, also to compare the treatment effects in TY with those of MBCT. The hypothesis was that all three groups, TY, MBCT and CBT would have similar effects on HRQoL with a possible advantage of TY and MBCT.

## Methods

### Settings and participants

This study was part of a randomized controlled trial investigating the effect of TY, MBCT and CBT, in patients on sick leave because of burnout caused mainly by difficulties at the workplace [18]. The intention with the larger study was to investigate how therapy in group with TY, MBCT and CBT worked for this kind of patients, from different aspects. The study was conducted at the Department of Neurobiology, Care Sciences and Society, Division of Family Medicine and Primary Care, at Karolinska Institutet, Stockholm, Sweden, from September 2007 until November 2009*.* Seven hundred and two people were assessed for eligibility. They came from different occupations and were recruited foremost from primary health care centres in the Stockholm County Council, as most patients with work-related disorders are found in primary care [10]. The trial ended due to time limitation.

**Fig. 1:**
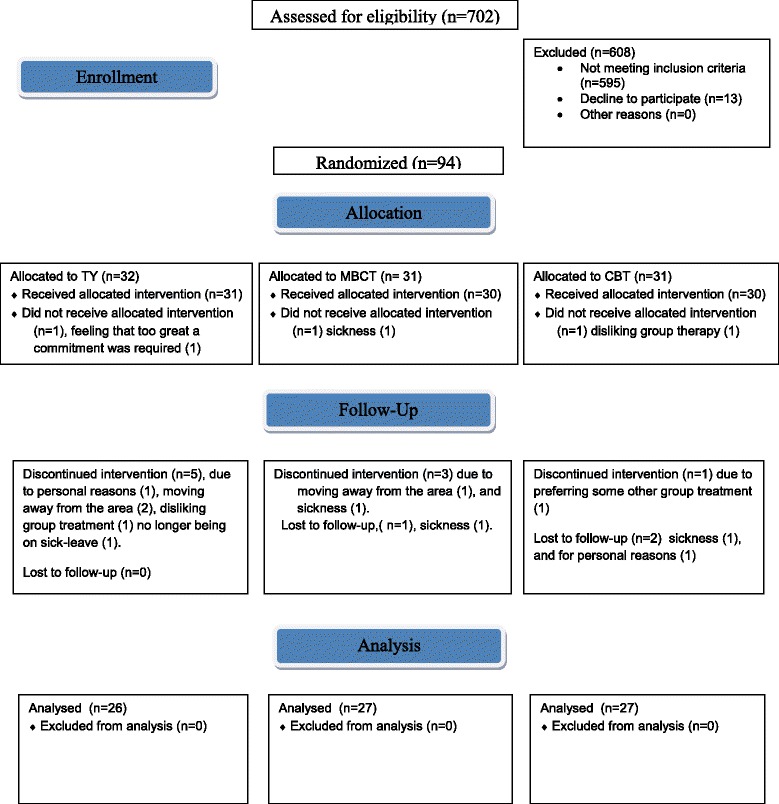
Flow diagram

Before inclusion, they underwent psychological examination by a physician (first author) and a licensed psychotherapist (second author) to confirm the diagnosis of Exhaustion Syndrome, and they were assessed for psychiatric co morbidity. The inclusion and exclusion criteria are listed in Table 2. All patients were on sick leave; 50%, 75% or 100%, prescribed by their ordinary physician and all received sickness benefits, that is, they received financial compensation for medically certified illness. After a baseline clinical examination and assessments (results presented elsewhere) by a physician, the participants were allotted to one of the three treatments. The HRQoL questionnaire was self-completed directly into the database before and after treatment, either at the time of the interview (before treatment) or later on via a computer link, using a personal code. Allocation to the treatment groups was done by block randomization, separately for women and for men, using sealed nontransparent envelopes, before the start of each treatment round. Generation of the random allocation sequence was prepared by an independent person and randomization was performed by the first author. One hundred and seven patients, 94 women and 13 men, fulfilled the inclusion criteria and were invited to participate. Thirteen of these one hundred seven patients declined to participate because they thought too great a commitment was involved or due to other personal reasons. Finally, 94 patients were included in the study, 84 women and 10 men. Please see flow chart, (Fig. 1). The assignment to interventions was blinded to those assessing outcomes by using a different code for each participant at the assessment.

**Table 2:** Inclusion and exclusion criteria

Inclusion critera: • Aged 18 through 65 years • Being on at least 50% sick-leave at the interview. Sick-leave for a maximum of one year if on full-time sick leave at the interview, or for a maximum of three years if on part-time sick leave at the interview • Body mass index (BMI) of between 18 and 26 • Meets the diagnostic criteria for Exhaustion Syndrome from the Swedish National Board of Health and Welfare, ICD 10 code; F43.8A	
Exclusion criteria: • Having other diseases that could give similar symptoms or hamper recovery • Not speaking Swedish well enough and not being well enough to participate in the study interventions • Using medication, including medication containing glucocorticoids. Exceptions: antidepressants, sedatives, contraceptives and hormone replacement therapy. The patients were asked to take the same prescribed doses at both assessment times.

### Dropouts

A total of 14 patients dropped out during the treatment (Fig. 1). Their subscale scores at baseline as well as age and gender did not differ significantly from those of the remaining participants.

### Interventions

All three groups, TY, MBCT and CBT received three hours of supervised group training per week and the participants practiced on their own for 1–1½ hours, 3–4 times a week, including homework, for a total of at least 7 h per week over five days. All participants followed this schedule, including the specific practices for each treatment arm stated below, for 20 weeks. All three groups practiced various practical skills, such as formulating a self-motivated day-to-day activity chart and planning and executing a micro-pause as homework, see below, which was assessed during the following week’s group session. The activity chart was used to help the patients create a realistic planning system. The preceding night they planned the next day’s activities, and then during the day they tried to follow their planned activities. In the evening they evaluated their work and rated how manageable their day had been and how they had felt emotionally during the day, thus gradually coming to understand their limitations; i.e. validation [43]. During the micro-pause they tried for several minutes to be present with their bodily sensations and emotions while doing some sort of brief practical task. The micro pause aims at helping the patients to come into closer contact with how they feel and how things are affecting them. The groups consisted of 9–11 participants and three rounds of groups went through the program. The instructor for all of the TY groups was a physician (first author), a trained TY teacher with many years of personal practice and of teaching TY. In the CBT groups a licensed psychotherapist (second author) with extensive clinical experience in the field conducted the treatment.

### Traditional yoga

The participants in this arm practiced TY, a variation of Ashtanga yoga, which is a form of yoga with gentle movements and postures (asanas), breathing exercises and meditation (Table 3). The TY which was used in this study is mild and does not demand too much effort, considering the physical and mental condition of the patients. The program was designed specifically for this group of patients, and the program was introduced in two steps due to their condition and because they did not have much pervious yoga experience (Table 3). The overall purpose of the TY intervention was to help participants attain increased awareness of their own bodily sensations and emotional feelings.

**Table 3:** Exercises in the Traditional Yoga treatment

Exercises Name in Sanskrit (Name in English)	Weeks 1–5	Weeks 6–20
Padahastasana (Standing forward bend)	X	X
Supta pawanmuktasana (Knee-lock pose)	X	X
Bhujangasana (Cobra pose)	X	X
Ardha Salambhasana (Half grasshopper pose)	X	X
Digapranam (Salutation)	X	X
Vajrasana (Diamond pose)	X	
Breathing exercise	X	X
Vipareet Karan Mudra (Inverted pose)		X
Matsyasana (Fish)		X
Yoga Mudra (Sitting, forward bend)		X
Tadasana (Mountain pose)		X
Kapal Bati (Bellow breathing)		X
Chakki Chalanasana (Grind grain)		X
Meditation	X	X

The components of TY were

1. Physical movements and postures (asanas); about 70%
2. Breathing exercises; active and passive, about 20%
3. Awareness: During all exercises, focus on feelings and bodily sensation was encouraged in order to understand and experience what was going on physiologically and emotionally; and,
4. Processing feelings by exercising the previous three components, so the individual gradually comes in contact with and becomes more aware of his or her feelings and emotions [33], and learns how to be present with and to experience difficult feelings and emotions instead of avoiding them.

### Mindfulness–based cognitive therapy

The MBCT intervention was designed with the aim to teach mindfulness and cognitive skills as a means to note distressing thoughts and feelings. They include: Be aware of and recognize one’s own bodily sensations, attending to distressing thoughts and feelings by holding them in the presence, and cultivating acceptance and self-awareness. These exercises possibly contributed to a kind of awareness of present-moment experience together with a compassionate, non-judgmental state. Additional skills, such as how to plan a satisfactory day by their own definition (by the help of a self-motivated day-to-day activity chart), plan and execute micro-pauses, and accept negative feelings without being overwhelmed by them, were also on the agenda. Participants were taught to focus more on the emotional part of their feelings and less on their thoughts when they were practising mindfulness. Homework consisted of a protocol for measuring/observing negative feelings, body-mind awareness exercises, a day-to-day motivated chart, and instructions to follow a special theme for the week. All assignments and homework were assessed and analyzed during the following week’s group session.

### Cognitive behavioral therapy

In addition to the day-to-day activity chart and the micro pause, the CBT program comprised different working components such as cognitive restructuring, applied relaxation technique, identifying the stressors, coping with stress, and how to reduce the experience of daily stress. The homework included a protocol for negative thoughts, relaxation, and a special theme for the week. Each session had a specific theme based on different cognitive and behavioral concepts and the theory behind the practical exercises, working material, and the specific homework assignment. Reports on the daily working chart were used for self–monitoring of day-to-day behavior and reactions. The structure of a therapeutic session included: formulating a commonly agreed upon work agenda for the group, a brief period of relaxation, reflections on the previous session, follow-up of the homework assignment, introduction of new themes, and preparation for the next homework assignment.

### Assessment

The outcome variable HRQoL was measured using the full version of a generic instrument, the Swedish health-related quality of life survey, 1.0 (SWED-QUAL) [44], which is well validated in the Swedish general population [45]. SWED-QUAL is developed from the American medical outcome study, as is the SF-36 [46]. SWED-QUAL is more extensive than SF-36, with several questions about social, cognitive and sexual functioning, aspects of life which are often affected in patients with burnout [18]. SWED-QUAL has been used in a wide range of research concerning both somatic and psychiatric conditions [19, 47]. It comprises 67 self-assessed questions about the present situation (now or the past week) grouped into 13 subscales, each with a separate index, from 0 (worst) to 100 (best possible). The subscales and descriptions are listed in Table 4. The questions are formulated negatively or positively, with four alternative answers such as No, not at all = 1, Yes, slightly = 2, Yes, fairly much = 3 or Yes, very much = 4. For some of the questions a Likert scale format is used with answers ranging from completely agree = 4 to completely disagree = 1. In addition, six questions about gender, age, having a partner or not, marital status, cohabiting and education level are included.

**Table 4:** Characteristics of subscales in SWED-QUAL 1.0

Subscale		No. of items	Description
Physical wellbeing			
	Physical functioning	7	Extent to which health interferes with ability to perform physical activities (e.g., heavy manual work, sports, climbing stairs, dressing)
	Satisfaction with physical functioning	1	Satisfaction with physical ability to do what wanted
	Pain, frequency and intensity	6	Pain frequency, intensity and interference with activities of daily life (ADL), sleep and mood
	Role limitation due to physical health	3	Extent to which physical problems interfere with ADL
Emotional wellbeing			
	Role limitation due to emotional health	3	Extent to which emotional problems interfere with ADL
	Positive affect	6	Is a happy person, felt liked, emotionally in harmony, much to look forward to
	Negative affect	6	Felt nervous, tense, down, sad, impatient, annoyed
Cognitive function		6	Concentration, memory, capacity to take decisions, confusion
Sleep		7	Problems with sleep initiation and maintenance, sleep adequacy and somnolence
General Health perceptions		8	Health: prior and current, overall rating of health, immune defense, health worries
Satisfaction with family functioning		4	Satisfaction with family life in terms of cohesiveness, amount of support and understanding, amount of talking things over, overall happiness with family life
Satisfaction with partner functioning		6	Relation to spouse (or person felt closest to) in terms of saying anything wanted, sharing feelings, feeling close, being supportive
Sexual functioning		5	Interest in sex, capability to enjoy sex, having orgasm(w), getting and keeping erection(m)

### Statistical methods

The sample size needed was estimated to be approximately 20–25 participants per group. The calculation was based on estimation, as when the study was designed there were no relevant studies in this field, and power analysis to determine the (effective) sample size was not possible. In a population (a cohort study) with diabetes type I and II [48], the sample size was 40 persons per group with a power of 80% and α = 0.05 based on a difference of 8 points on the SWED-QUAL subscale “general health perceptions,” which is often used for comparison between groups and for sample size calculations. Since the participants in our study had a condition that was far worse than in the diabetic group (data not shown), we assumed that the sample size could be smaller.

Wilcoxon’s rank sum test [49] was used for comparison of the treatment groups’ subscale scores at baseline and for comparison of the between-group treatment effect. Wilcoxon’s sign rank was used for comparison of treatment effects [49]. Effect size was calculated using Cohen’s D [50]. Effect size is the ratio of the mean change in scores to the weighted SD and measures the relative effect of the treatment, that is, whether the results are clinically relevant or not. Cohen’s D < 0.2 is considered without importance, 0.2–< 0.5 is a small effect, 0.5–< 0.8 is a moderate effect and ≥0.8 is a large effect. When Cohen’s D is 1.0 the mean effect corresponds to the SD. The median and the confidence interval were determined using the Bonett-Price calculation [51]. The significance level was set to < 0.05. Because there were multiple comparisons, the Holm-Bonferroni correction was used in the statistical calculations for the outcome variables [52]. Missing data were found, with few exceptions, in the subscales sexual function and partner functioning. This is in accordance with previous studies [53]. Those who did not answer these items lived almost exclusively alone and had reported that they did not have a partner.

The analyses were carried out according to the protocol. Data analyses were conducted using the statistical program STATA 11.2 StataCorp. (2009). Stata Statistical Software: Release 11. College Station, TX: StataCorp LP.

## Results

**Fig. 2:**
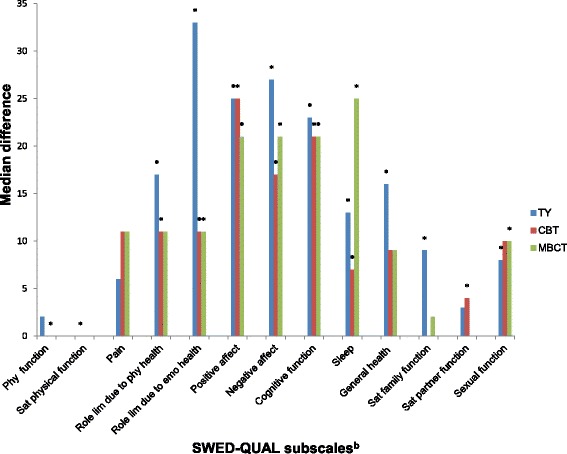
Test of treatment effect (post-pre)^a^ in SWED-QUAL^b^ median, subscales scores, ranging from 0 (worst) to 100 (best) possible, after 20 weeks treatment with Traditional Yoga (TY), Cognitive Behavioral Therapy (CBT) (control) or Mindfulness-based Cognitive Therapy (MBCT). Significant *p*-values, *P* < 0.05, and after Holm-Bonferroni correction are indicated by (*).Legend: ^a^ Wilcoxon’s sign-rank test was used for comparisons in each group ^b^ Swedish Health related Quality of Life Questionnaire

We have previously shown that these patients on sick leave because of burnout, mainly due to work-related causes, have markedly lower HRQoL in general [18], but randomized control studies measuring HRQoL after treating these severely sick patients are scarce. In this study, HRQoL was measured in the above-mentioned group of patients after a 20-week group treatment with TY (26 patients), MBCT (27 patients), or CBT (27 patients). The results are presented in Tables 6 and 7, and Fig. 2.

The majority of the patients were women, 81% in the TY group, 82% in the MBCT group, and 93% in the CBT group. All three groups had a high level of education (Table 5). Eighty percent of the participants in the study reported that they were on sick -leave with work-related causes. The occupations were white collar (47%), school and nursery (17%), blue collar (17%), health care (14%) and other (6%). All patients fulfilled the diagnostic criteria for anxiety and depression. There were no baseline differences between the treatment groups regarding sociodemographic variables (Table 5) or baseline scores in the 13 subscales constituting SWED-QUAL (Table 6). At baseline all subscales scores, except “physical functioning” was markedly low and were significantly lower compared to a healthy group who were working full time [18].

**Table 5:** Socio-demographic data among the study groups compared to the Swedish population

	Treatment group			Swedish population^a^
	TY	MBCT	CBT	
Sociodemografic data, n	All [men] *n* = 26[5]	All [men] *n* = 27[3]	All [men] *n* = 27[2]	All
Age, years, means ± SEM	43.4 ± 1.7 [43.6 ± 4.6]	41.3 ± 1.7 [45 ± 6.7]	47.2 ± 1.5 [44.0 ± 3.0]	
Sick-leave % ± SD	88.3 ± 21.0 [83.3 ± 25.8]	85.5 ± 19.1 [75.0 ± 25.0]	78.2 ± 23.9 [50.0 ± 0.0]	
Body mass index (BMI) ± SD	22.6 ± 2.4 [23.9 ± 3.0]	22.4 ± 2.0 [23.2 ± 2.9]	22.9 ± 1.9 [24.4 ± 1.4]	
Education, years, n (%)				(%)
≤ 9 years	0(0)	1(4)	1(4)	(17)
> 9–12 years	9(35)	9(33)	5(18)	(46)
> 12 years	*17(65)*	*17(63)*	*21(78)*	(36)
Having a partner, n (%)	17(65)	16(59)	16(59)	
Medication^b^, psychotropic drugs prescribed, n	7	11	12	~ (10)

^a^The official Swedish population statistics for 2003–2008 from The National Board of Health and Welfare, The National Social Insurance Board and

Statistics Sweden are included for comparison in this table. ^b^ATC code NO6, antidepressants; ATC code N05, sleep medication and tranquillizers and ATC code NO2, pain killers

**Table 6:**
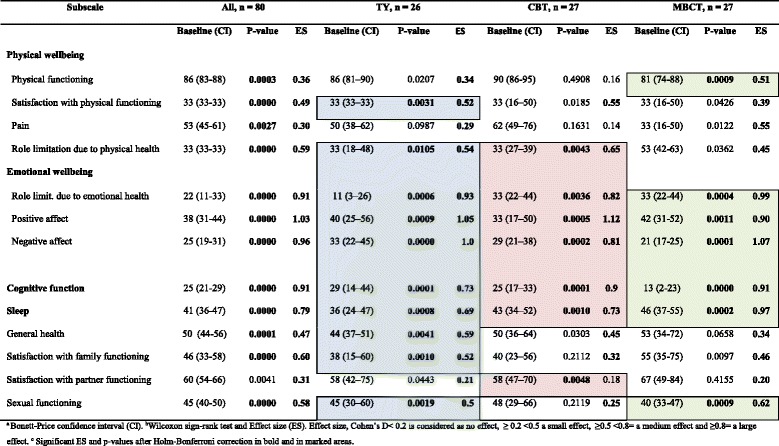
SWED-QUAL baseline median subscale scores ranging from 0(worst) to 100(best possible)^a^, and test of treatment effect^b^ per group and for all. Main results marked^c^

### Effects of treatment

All three groups had a good effect on HRQoL in general as measured by SWED-QUAL, with the highest numerical increase (Fig. 2) in subscales related to the main domains affected in burnout [54]. These include emotional well-being, cognitive function, sleep and, to a lesser degree, physical well-being (Tables 4 and 6), with a median increase of 15%–167% in most of the significant subscales. The effect size in these subscales was, with one exception, medium or large (Table 6). When an overall analysis was made, comprising all three groups together, *n* = 80, twelve out of thirteen subscales showed a significant change after Holm-Bonferroni correction (*P* < 0.05) and Cohen’s D was medium or large in the subscales related to the main domains affected in burnout, as mentioned above (Table 6).

### Traditional yoga

Yoga has previously not been used as a treatment in this group of patients, but might have a good effect, as TY employs exercises involving the main domains affected in burnout. The group treatment effect measured as pre/post scores showed an improvement in HRQoL in general with high median numerical differences in most of the 10 statistically significant subscales (Fig. 2). The largest improvements were seen in the subscales concerning emotional well-being (Tables 4 and 6), with subscales such as “role limitation due to emotional health”, *P* = 0.0006; “positive affect”, *P* = 0.0009; and “negative affect”, *P* = 0.0000; physical well-being (Tables 4 and 6), with the subscales “satisfaction with physical functioning”, *P* = 0.0031; and role limitation due to physical health”, *P* = 0.0105; together with the subscales “cognitive function,” *P* = 0.0001 “sleep”, *P* = 0.0008 and “general health.” *P* = 0.0041 (Tables 4 and 6). Median differences ranged from 13 to 27 points, with a percent increase ranging from 34% to 138%, in these subscales. The largest percentage increases were, as expected, found in subscales concerning emotional well-being, with an increase of 138% in the subscale “role limitation due to emotional health” and an increase of 100% in the subscale “negative affect.” There was a discrepancy in the effect of the treatment between the subscales in physical wellbeing; on the one hand “physical functioning,” 2 points (3% increase) and “pain,” 6 points (12%increase), both non-significant and, on the other hand “role limitation due to physical health”—a 17-point median difference (55% increase), and “satisfaction with physical function”, 0 points (0% increase) but both significant, which was a surprise. Another discrepancy was seen in TY. While there was a significant effect in both “satisfaction with family function,” *P* = 0.0010, 9 points (27% increase), and “sexual function;” *P* = 0.0019,8 points (18% increase), the “satisfaction with partner functioning was non-significant.The clinical effect, measured by Cohen’s D, was seen in all of the 13 subscales in TY, and there was a medium or a large effect size in all 10 statistically significant subscales for the TY group, which was quite expected, as the TY treatment covers many different aspects.

### Mindfulness–based cognitive therapy

In the MBCT group the effect of the intervention measured by SWED-QUAL, showed an increase in seven of the 13 SWED-QUAL subscales, with high significant median differences *P* < 0.05 (Fig. 2) after Holm-Bonferroni correction. The largest increase in scores was found in the subscales “sleep”, *P* = 0.0002, “cognitive function”, *P* = 0.0000 (Tables 4 and 6) and in emotional wellbeing with the subscales “negative and positive affect”, *P* = 0.0001 and *P* = 0.0011 (Tables 4 and 6). All which are important factors in burnout, and possible indicators of positive change in a patients’ quality of life. Calculation of the median numerical difference of each subscale pre and post treatment showed median differences between 10 and 25 points (Fig. 2) with an increase in subscale scores ranging from 25% to 167% in all seven of the significant subscales, except for “physical functioning.” The largest percent increase was found in the subscales “cognitive function” 167%, “negative affect” 120% and “positive affect” 64%. The effect size was medium (≥ 0.5) or a large (≥ 0.8) in all of the subscales which showed a statistical improvement *P* < 0.05 (Table 6). Also in MBCT the subscales in the domain physical wellbeing were not consistent. The subscale “physical functioning” had, a significant increase in median scores, *P* = 0.0009, 0 points (0% increase). Additionally, “role limitation due to physical health” and the subscale “pain” had an 11 point difference in scores (21%increase) in both. Although they are not statistically significant according to Holm-Bonferroni correction, they showed a medium and small treatment effect respectively. In MBCT “sexual functioning” was significant, *P* = 0.0009, with a medium effect size, while the subscales “satisfaction with family function” and “satisfaction with partner functioning” were non-significant, both with a small effect size.

### Cognitive behavioral therapy

The results here show a similar pattern that was seen in the MCBT group, with high median numerical differences in most of the 7 statistically significant subscales (Fig. 2). The largest increases in scores were found in subscales concerning important domains in burnout such as emotional well-being (Tables 4 and 6) with the subscales “role limitation due to emotional health”, *P* = 0.0036, “positive affect”, *P* = 0.0005 and “negative affect”, *P* = 0.0002; physical well-being (Table 4) with the subscale “role limitation due to physical health”, *P* = 0.0043; and the subscales “cognitive function”, *P* = 0.0001 and “sleep”, *P* = 0.0010. Median differences ranged from 7 to 25 points in these subscales (Fig. 2), and with an increase of 15% to 100%. The largest percent increase was found in the subscales “positive affect” (100%) and “cognitive function” (80%), both important domains affected in burnout. The clinical effect, measured by Cohen’s D, was medium or large in six of these seven subscales, which indicates a good clinical effect (Table 6). Despite an overall large effect on HRQoL and effect on “role limitation due to physical health,” 11 points (34% increase) no effect was seen in the subscale “physical function,” 0 points (0% increase) or in “pain”, 11 points (18% increase), which was quite surprising (Table 6). In CBT “satisfaction with partner functioning” was significant, *P* = 0.0048, but without effect size. In “satisfaction with family functioning” and “sexual functioning” a non-significant effect was found with a small effect size for both.

In all the treatments, some subscales showed a low, or no numerical median difference, but the *p*-value showed that the difference was significant, and/or a Cohen’s D indicated a clinical effect (Table 6). This was because both parametric and nonparametric methods for analyzing the treatment effects were used.

### Comparison of the treatments

The overall effect from TY and MBCT were slightly better than that from CBT measured by Cohen’s D (Table 6). When calculating effect size for the treatment effects, a small difference was found in favor of TY in five of the subscales, with a Cohen’s D of 0.22–0.43 (Table 7), and in favor of MBCT in seven of the subscales, with a Cohen’s D of 0.32–0.44 (Table 7). This indicates a slightly better effect from TY and MBCT than from CBT in certain domains. Both TY and MBCT had a larger increase in “negative affect,” compared to CBT, *P* = 0.09 and *P* = 0.19, which, we assume, in turn helped to increase “role limitation due to emotional health” more in TY and MBCT compared to CBT, *P* = 0.25 and *P* = 0.36. Also, measured numerically (12 subscales) (Fig. 2), and measured by percentage increase (6 subscales), (data not shown), TY showed a larger increase (14%–69%) than did CBT, although not a statistically significant one. In five subscales the CBT group improved more than the TY group did, but to a lesser degree (4%–49%), (data not shown). When measured by percent increase (7 subscales), (data not shown), MBCT showed a larger increase (2%–87%) than did CBT. In four subscales the CBT group improved more than the MBCT group did, but also to a lower extent (5%–36%), (data not shown). Finally, when comparing the effects of TY and MBCT, the overall effect from TY was slightly better but not significant when comparing median, numerical increase (Fig. 2). The effect from TY and MCBT were similar regarding effect size, with a better effect for both treatments in two subscales each, with a small effect size. When measured by percent the increases were similar (data not shown). Six subscales in TY showed a larger increase (3%–88%) than did MBCT, and MBCT showed a larger increase in five subscales (2%–121%), (data not shown), than did TY.

**Table 7:** Comparison between the groups’ treatment effects, measured by SWED-QUAL subscale scores, as *p*-values^a^ and effect size^b^

Subscale	TY - CBT		MBCT - CBT		TY - MBCT	
	*P*-value	ES	*P*-value	ES	*P*-value	ES
Physical functioning	0.28	0.09	0.13	**0.44 M**	0.70	**0.29 M**
Satisfaction with physical functioning	0.82	0.06	0.89	−0.08	0.71	0.06
Pain	0.39	0.18	0.38	−0.07	0.66	0.13
Role limitation due to physical health	0.76	−0.02	0.47	**0.33 M**	0.75	0.04
Role limitation due to emotional health	0.25	**0.39 Y**	0.36	**0.32 M**	0.95	0.1
Positive affect	0.94	0.07	0.52	−0.17	0.70	**0.23 Y**
Negative affect	0.09	**0.43 Y**	0.19	**0.37 M**	0.65	0.09
Cognitive function	1.0	0.01	0.38	**0.32 M**	0.35	**0.28 M**
Sleep	0.40	0.16	0.16	**0.36 M**	0.66	0.16
General health	0.41	**0.26 Y**	0.88	0.04	0.69	**0.20 Y**
Satisfaction with family functioning	0.14	**0.22 Y**	0.32	0.15	0.46	0.07
Satisfaction with partner functioning	0.60	0.05	0.47	0.01	0.87	0.04
Sexual functioning	0.39	**0.31 Y**	0.48	**0.30 M**	0.91	0.09

^a^Wilcoxon rank-sum test. ^b^Effect size (ES), Cohen’s D < 0.2 is considered as no effect, ≥ 0.2 < 0.5 a small effect,

≥0.5 < 0.8 = a medium effect and ≥0.8 = a large effect. Significant ES and *p*-values after Holm-Bonferroni correction in bold

### Satisfaction with the treatment

All patients reported good effect from, and satisfaction with, the received treatment. This was despite the fact that they were assigned the treatments and had, in the interview, in many cases reported that they preferred another of the treatments.

### Attendance, adherence and adverse events

Attendance at all three groups’ sessions was recorded by the yoga teacher or the therapist. Homework was reported via the activity chart executed every day, indicating what exercises they had performed. Homework including activity chart was turned in to the yoga teacher or therapist each week.

The average attendance for the participants was not significantly different between TY mean 69%, MBCT mean 75%, and CBT mean 70%. Adherence: Homework was practiced in the TY mean 89.4 h, in the MBCT group 90.4 h, and 83 h in the CBT group. The majority of the absences were due to tiredness or unexpected events. The therapist in the MBCT and CBT groups reviewed the content of the missed session and homework at the next session. If absent more than one session the therapist or the yoga teacher contacted the participant to keep the participant engaged and to maintain program continuity.

Adverse events: The yoga teacher or therapist queried participants regarding potential problems and adverse events at each session, and these were recorded. The participants were also encouraged to contact the therapist/yoga teacher regarding any potential concerns. No serious treatment-related adverse events were reported.

## Discussion

In this randomized controlled trial we have shown that a 20-week group treatment with two new treatments; TY and MCBT, or CBT (control), improved HRQoL in severely sick patients on sick leave because of burnout, which supports our hypothesis. The patients were sicker than most patients in earlier studies and they had in most cases been sick for a longer time at inclusion, than patients in previous studies. The treatments had a good effect in general on HRQoL as measured by SWED-QUAL and the effects were larger than we had expected. Effect sizes were medium or large in all the significant subscales in all three groups, except for one in CBT. Comparison between the treatments showed a slightly larger effect sizes for TY and MBCT, in comparison to CBT, in several of the subscales.

### Emotional wellbeing

The large impact on emotional well-being, one of the main components in burnout, was seen in all three subscales, “positive affect,” “negative affect” and “role limitation due to emotional health”(Tables 4 and 6). The improvement in TY may be caused by the mild physical movements, the rhythmic, regular breathing and the constant awareness training during the exercises [33, 55, 56]. These are known to have an effect on both the parasympathetic and the sympathetic nervous systems, as well as on positive and negative affect (emotions) [57, 58]. Also, the results in the TY group might have benefitted from employing exercises in the physical, emotional and cognitive domains simultaneously. The MBCT treatment targets mostly the cognitive and emotional part of the individuals’ experience (Segal et al., 2002).. It is found that patients who suffer from stress, anxiety and depression often use the strategy of ‘experiential avoidance’. This strategy is the alteration of frequency of thoughts, feelings, bodily sensations, or memories. Although these alterations may not bring any real comfort or lessen the severity of suffering, those practices end up being even more costly, ineffective and damaging to the patients habitually using it. One of the ways to encourage patients to replace experiential avoidance is with mindfulness acceptance. This is to accept real experiences, emotions and thoughts as they are. It is an intentional behavior that alters the function of inner experiences from events to be avoided, to a focus on interest, curiosity, and observation as part of living a valued life. MBCT has previously show good effect on burnout in a group of primary care physicians [59] and mindfulness has shown to be effective in primary care patients suffering from stress related disorders [60]. In CBT treatment the improvement might be due to cognitive changes caused by the use of different kinds of methods to detect negative thoughts and to replace them with more positive ones. This may in turn have an effect on “positive” and “negative affect.” Previous studies have shown that CBT has a good effect on positive and negative affect [61].

### Cognitive function

Emotion is also known to be closely connected to cognitive function [62, 63], and may affect emotional wellbeing, and vice versa. Cognitive function, which signifies concentration ability, understanding capacity, memory and the capacity to focus on day-to-day life, is an important component of burnout. The improvement in scores in “cognitive function” is similar, although TY treatment uses different methods of cognitive training, from MBCT and CBT. This indicates that TY might be helpful for people who, for various reasons, find it difficult to participate in CBT or MBCT. Cognitive function has previously been shown to increase after six weeks of Yoga training in breast cancer patients [64], and in menopausal women after a Yoga program [65]. Also, in a group of elderly participants cognitive function increased together with emotional wellbeing after following a mindfulness program [66]. Numerous studies have shown a good effect on different aspects of cognition [73, 76], which supports our findings.

### Physical wellbeing

The subscale score at baseline for “physical functioning” was comparatively high in all three groups; probably due to their relatively young age and that they were previously healthy. The results are in accordance with earlier findings in a group of persons experiencing emotional ill-being and attending a mind-body-medicine course [67]. Furthermore, previous findings have shown that emotional and cognitive functions are much more readily affected than physical function in a stressful situation [68].

The subscales concerning physical wellbeing showed inconsistent patterns in the three groups with betterment in some subscales but not in others, despite the increase in emotional wellbeing. Burnout patients are generally inclined to negative affect and consistently report declining health symptoms, such as mental health and somatic complaints [69] Furthermore, negative affect has a weak and inconsistent association to objectivity in regards to assessed health status [70]. Suner-Soler et al. (2013) finds that high levels of burnout especially in the “emotional exhaustion” component, leads to deterioration in the health-related quality of life both physically and mentally [71]. And recent evidence suggests that burnout also has a negative impact on physical health [72]. Thus, we expected that along with the improvement in emotional wellbeing there should be a simultaneous improvement in physical wellbeing. In TY treatment, bodywork in the form of mild yoga postures and movements might give the participants a more accurate idea about their level of functioning. Here, in TY, the subscale “physical functioning” was non-significant while “role limitation due to physical health” showed a significant increase. A possible explanation might be that while “physical functioning” also contains questions on heavy work and strenuous exercises, the subscale “role limitation due to physical health” concerns day-to-day activities that a person usually performs anyway and an improvement here may probably be experienced earlier. In a previous study examining the effects of mild yoga it was found that 83% of the yoga practitioners improved in overall physical function and capacity [73], but these patients were older and at risk for cardiovascular disease. The MBCT group showed the opposite pattern, with a significant increase in the subscale “physical functioning” but a non-significant increase in the subscale “role limitation due to physical health”. This difference we assume exist because of the already higher, baseline median score, and therefore the subscale “role limitation due to physical health” do not show a significant increase. The significant increase of “physical functioning” could be counted as a positive sign for the burnout patients. In the CBT treatment group the median subscale score for “physical functioning” was high at baseline, but despite the increase in “role limitation due to physical health,” emotional well-being, cognition and sleep the subscale score for “physical functioning” actually did not change. CBT treatment comprised relaxation as a means of increasing body awareness in addition to the cognitive exercises and strategies, but this was apparently not enough to counteract the influence of burnout over time. Also, the scores were high from the beginning, which might have made further improvement in the scores less likely. These findings contradict previous research showing that CBT improved physical function in patients on sick leave for stress-related disorders as measured by SF-36, a HRQoL questionnaire similar to SWED-QUAL. However, in that study the participants’ baseline scores were not reported [74]. The low, non-significant improvement of the subscale “pain”, in all three groups was a surprise. Also, there was only a low effect size in TY and no effect size at all in CBT, while MBCT had a medium effect size. Pain is often one of the first symptoms experienced in stress related disorders, and we expected somehow that there should be an improvement after treatment. But, as pain is an unspecific symptom, it might be the other way around; pain may be the last symptom that disappears. Here, MBCT had a significant increase in “physical functioning” which might have influenced the improvement in the subscale “pain”, while in TY the low increase might be caused by the bodywork which made them more in contact with their body. This might have made them experience, and assess their pain to a larger extent.

### Sleep

The subscale sleep showed good results in the all three groups group. In TY this impact on sleep may be explained by a combination of the relaxing and overall stress-reducing effect together with increased calmness, lowered negative affect and improved body awareness, which TY is reported to have [75]. In previous studies yoga has also been shown to have a good effect on sleep disturbances [73], which is in line with our results. The subscale “sleep” was the one most improved for the MBCT group, and that improvement would have helped reduce the severity of burnout, as previous studies showed that insufficient sleep predicts burnout [76]. An eight week treatment containing mindfulness meditation has previously shown an effect on chronic Insomnia in a group of adults [77], and MBCT improved polysomnographic and subjective sleep profiles in antidepressant users with sleep complaints [78], which support our findings. In CBT, which is a common therapy used for sleeping problems, showed good effect on insomnia in a group of depressed patients ([79, 80].

The differences between the groups regarding the subscales “satisfaction”.

The improvement in sexual function seen in the TY group was probably because of the increase in both emotional and physical well-being. The result is in accordance with studies by Dhikav et al. [81, 82]. In MBCT the non-significant improvements in “satisfaction with partner functioning” and “satisfaction with family functioning” contrasted to “sexual functioning” which had a significant effect in MBCT. This result evoke an interesting question, how can sexual functioning score significantly higher for MBCT when satisfaction with family functioning and partner functioning scored low? Wändell et al. found psychiatric conditions would have been the second most important predictor, next to age of sexual dysfunction in diabetic patients [53]. Fernros et al., also found that physical functioning had a strong correlation with sexual functioning [67]. In this study, physical functioning, emotional health and cognitive functioning are all significantly high for participants in MBCT, and thereby concluding that, a low score in partner and family functioning may not necessarily affect sexual functioning.

The trend for TY and to some extent the trend for MBCT over CBT in the subscale “negative affect” might indicate that these two treatments have an additional effect compared to CBT. This is in line with our quest but more studies are needed to explore this.

Although a general increase in HRQoL is not necessarily followed by an increase in work ability, an improvement in the scores of subscales “role limitation due to emotional health” and “role limitation due to physical health” may indicate an increased capacity to work. These two subscales deal with how our role functions in various professions are affected by how one feels emotionally and physically. All three treatment groups, TY, MCBT and CBT had large improvements in these subscales. Previous research has not been able to show an increased work ability with CBT as a stand-alone treatment, but usually these treatments are shorter (8–16 weeks) [28].

When the scores in the TY, MBCT and CBT treatment were compared with those of a healthy group working full time [18], all three groups showed decreased median differences in scores after treatment compared to the healthy group. In ten subscales in the TY group, in seven subscales in the MBCT group and in five subscales in the CBT group, the difference in subscale score between the treatment groups and the healthy group were no longer significant after median regression (data not shown), which indicates a good effect of the treatment.

As the data are ordinal, group-wise and between-group analyses of scores were done using nonparametric methods. For the sake of comparison with previous studies, percent increase and Cohen’s D were calculated. An analysis with parametric methods was also carried out, and it yielded the same results concerning *P*-values. The results pre- and post treatment differ slightly when presented as means instead of medians, but the general picture remains. Another phenomenon, “regression towards the mean,” might have influenced the scores. For example, if SWED-QUAL is filled in a second time in close approximation to the first occasion, the scores tend to be closer to the mean. This might have influenced the scores to some extent in our group, but it is unlikely to have changed the results.

### Strengths and limitations

One of the strengths of this study is that it is a RCT, and that the study group was a clinical sample of patients with different occupations, reflecting the situation of these patients on sick leave in occupational medicine and in primary care. Also, they were diagnosed with Exhaustion Syndrome beside self-assessment. Furthermore, HRQoL was measured by SWED-QUAL, which comprises questions covering most components known to be affected in burnout. Also, SWED-QUAL has been shown to be stable over time, so that a change in scores reflects a real change [45]. The interventions were group treatments, which may facilitate implementation in the health care system and as prevention at work sites and companies. The limitations were the small group size and that there were few men, although it reflects the actual situation. All the patients had actively applied to participate in the study, which reflects initiative and a certain level of strength, and thus patients with less motivation and strength are not represented. Also, all participants were receiving sickness benefits, and therefore persons outside the labor market were not included in the study. In addition, many of the participants increased their level of activity during the study. This in turn resulted in reported increased symptoms and less self-reported improvement, which may have negatively influenced the SWED-QUAL subscale scores and thus underestimating the effects of the treatment. The therapies in the study were provided by the first and second authors, which may have influenced results. Another limitation is that the restricted sample size did not allow sub analyses based on factors such as gender, age, different professions or level of sick leave. Although some individuals reported that the stressor inducing the symptoms leading to exhaustion syndrome was their personal situation, we do not know if the situation at work also contributed to the symptoms developing, as we did not ask for this information specifically. Despite the improvement in HRQoL, we do not actually know whether the participants’ levels of burnout decreased or not. In summary, this study did not have the basic foundation to judge whether TY, MBCT and CBT treatments certainly decreased the suffering of the burnout patients. Nevertheless, it showed that TY, MBCT and CBT as group treatments positively influenced several subscales of SWED-QUAL for patients with burnout who were on sick leave.

### Implications for health care and research

We assume that the results are generalizable to patients undergoing rehabilitation in occupational medicine and primary care settings, where most of these patients are found, despite the limitations of inclusion and exclusion criteria such as age, gender, whether working or not, under- or overweight and having other diseases. Also, we believe that the results are generalizable to burnout patients on sick leave in other health care settings such as psychiatric, internal or emergency medicine, as patients initially might seek care outside of occupational medicine and primary care due to the wide variety of symptoms. More therapies, with different working components, could help patients improve their HRQoL and decrease the risk of future morbidity, both as early intervention and in severe cases, as in this study. All three group treatments can be used as health promotion and burnout prevention in occupational health care management in companies and, after the active treatment phase is over, to prevent relapse. This is an advantage, as these can help patients handle stress at work and in their day-to-day lives.

Future research with larger groups, effective sample size determination, and with equal representation of both genders is needed to confirm the results from this study and to further explore the efficacious components in each of the treatments. Also, studies to explore whether increased HRQoL decreases burnout and improves return-to-work rates after longer treatment with TY, MCBT and CBT would be beneficial, as well as studies which combine TY, MCBT and CBT with workplace intervention methods. A follow-up study is planned to evaluate the long-term effects together with the relapse ratio and cost-effectiveness calculations.

## Conclusions

Group treatment with TY, MBCT or CBT had large equal effects on HRQoL, specifically on several main components such as emotional well-being, cognitive function and sleep, in severely sick patients on sick leave because of burnout. Also, but to a lesser extent, a treatment effect on the physical component was seen in TY. Comparisons between groups showed no significant differences in scores, but a small difference in effect size in favor of TY in five subscales and in MBCT in seven subscales when compared with CBT. Our results indicate that all three group treatments could be used in rehabilitation in different parts of the health care system to increase HRQoL and, as such, lower the risk of future morbidity in patients on sick leave because of burnout. The results can also be used for power calculations in future research.
